# Impact of SGLT2 Inhibitors on Very Elderly Population with Heart Failure with Reduce Ejection Fraction: Real Life Data

**DOI:** 10.3390/biomedicines12071507

**Published:** 2024-07-07

**Authors:** Jorge Balaguer Germán, Marcelino Cortés García, Carlos Rodríguez López, Jose María Romero Otero, Jose Antonio Esteban Chapel, Antonio José Bollas Becerra, Luis Nieto Roca, Mikel Taibo Urquía, Ana María Pello Lázaro, José Tuñón Fernández

**Affiliations:** 1 Cardiology Department, Fundacion Jimenez Diaz Universitary Hospital, 28040 Madrid, Spain; 2 Cardiology Department, Son Spases Universitary Hospital, 07120 Palma, Spain; luisnietor93@gmail.com

**Keywords:** HFrEF, elderly, SGLT2i, propensity score

## Abstract

(1) Background: The validation of new lines of therapy for the elderly is required due to the progressive ageing of the world population and scarce evidence in elderly patients with HF with reduced ejection fraction (HFrEF). The purpose of our study is to analyze the effect of SGLT2 inhibitors (SGLT2i) in this subgroup of patients. (2) Methods: A single-center, real-world observational study was performed. We consecutively enrolled all patients aged ≥ 75 years diagnosed with HFrEF and for treatment with SGLT2i, and considered the theoretical indications. (3) Results: A total of 364 patients were recruited, with a mean age of 84.1 years. At inclusion, the mean LVEF was 29.8%. Median follow-up was 33 months, and there were 122 deaths. A total of 55 patients were under SGLT2i treatment. A multivariate Cox logistic regression test for all-cause mortality was performed, and only SGLT2i (HR 0.39 [0.19–0.82]) and glomerular filtration rate (HR 0.98 [0.98–0.99]) proved to be protective factors. In parallel, we conducted a propensity-score-matched analysis, where a significant reduction in all-cause mortality was associated with the use of SGLT2i treatment (HR 0.39, [0.16–0.97]). (4) Conclusions: Treatment with SGLT2i in elderly patients with HFrEF was associated with a lower rate of all-cause mortality. Our data show that SGLT2i therapy could improve prognosis in the elderly with HFrEF in a real-world study.

## 1. Introduction

Heart failure (HF) remains a disease of increasing prevalence and morbidity, affecting at least 64 million people worldwide [1]. The validation of new lines of therapy for those in older adulthood is required, due to the increased morbidity and mortality of this subgroup of people and the progressive ageing of the world population. It is therefore essential to assess efficacy and tolerability, which may be of particular concern in this subgroup, not only because of advanced age, but also because of polypharmacy, among other factors [2,3]. As a result, these patients have a higher proportion of other comorbidities, and more than 70% of HF patients 80 years or older fulfill the frailty criteria [4]. All these factors may also lead to the underutilization of new lines of treatment in older patients with HF. On the other hand, two SGLT-2 inhibitors (SGLT2i), empagliflozin and dapagliflozin, have proven to be beneficial in patients with chronic HF with reduced ejection fraction (HFrEF), with or without type 2 diabetes mellitus (T2DM), in the EMPEROR-Reduced and DAPA-HF studies [5,6]. Currently, the European Society of Cardiology guidelines recommend SGLT-2 inhibitors in people with HFrEF [7]. Nevertheless, data on the role of SGLT2i in elderly patients with HFrEF remain scarce. The purpose of our study is to analyze the effect of SGLT2i in this subgroup of patients.

## 2. Materials and Methods

### 2.1. Patients and Study Design

We conducted a single-center, real-world observational study. We consecutively enlisted all patients aged ≥75 years diagnosed with HFrEF (defined as an ejection fraction <40%) for treatment with SGLT2i from November 2019 to November 2022, and considered the theoretical indications. A specific database compiled in the cardiac imaging department of our center was used to screen for patients meeting the criteria. All patients received regular medical supervision according to their symptoms, and the guidance of their physician (cardiologists or general practitioners) to optimize treatment. Investigators reviewed each patient’s electronic medical records in order to collect clinical, electrocardiographic and echocardiographic data. The study design and protocol have been revised and approved by the Clinical Research Ethics Committee of our institution (Ref. EO093-18 FJD). This investigation was carried out in accordance with the principles outlined in the Declaration of Helsinki.

### 2.2. Outcomes and Follow-Up

The outcomes analyzed in our study were the rate of all-cause death and major cardiovascular events. Here, cardiovascular events include death of any cause or admission due to HF. HF admission was defined as admission to a healthcare facility lasting > 24 h due to the worsening of HF symptoms, and followed by specific treatment for HF (regardless of the cause of decompensation). Clinical events and death during follow-up were collected from patients’ electronic health records or, if not available, from telephone interviews with patients or relatives.

### 2.3. Statistical Analysis

Data were subjected to descriptive statistical analysis via frequency measurements (absolute frequencies and percentages) for qualitative variables, and mean and standard deviation were used for quantitative variables. The magnitude of the effects of the variables was expressed in the form of hazard ratios (HRs) and 95% confidence intervals (CIs). A univariate analysis of the quantitative variables was performed using the Student t test when the variables were normally distributed and the Mann–Whitney U test when the distribution was not normal. The qualitative variables were analyzed using the χ^2^ or the Fisher exact test. Because observational studies do not allow for randomization, we planned two different approaches in order to avoid potential confounding factors: multivariate Cox proportional hazard and propensity-score (PS)-matched analysis. These two analyses were used to determine significant predictors of cardiovascular events and mortality.

First, we performed a multivariate analysis with Cox (backward stepwise) regression. Of all the baseline variables collected, we selected those with the potential to act as confounding factors. The selection criteria were as follows: first, clinical and biological plausibility and, second, the statistical criterion of Mickey, excluding all those variables that returned a *p* value > 0.20 on univariate analysis [8]. The variables included in the multivariate Cox regression analysis for mortality were as follows: age (continuous variable), gender (male/female), glomerular filtration (continuous), cerebrovascular disease (yes/no), rhythm (sinus/non sinus), QRS > 120 msec (yes/no), ARNI (yes/no), beta-blockers (yes/no), SGLT2i (yes/no), and LVEF > 50% at follow up (yes/no). In the case of cardiovascular events, they were as follows: age (continuous variable), gender (male/female), glomerular filtration (continuous), frailty (yes/no), cerebrovascular disease (yes/no), NYHA class (I–II/III–IV), cardiac resynchronization therapy (yes/no), ACEi/ARB (yes/no), beta-blockers (yes/no), ARNI (yes/no), andSGLT2i (yes/no).

Second, we performed a PS-matched analysis. The PS was calculated with an ordered logistic regression model, taking the SGLT2i group as the dependent variables and adopting a parsimonious approach. In the first step, all the following variables were included in the univariate analysis: age, gender, diabetes mellitus, hypertension, glomerular filtration, chronic lung disease [asthma, chronic obstructive pulmonary disease (COPD) or sleep apnoea hypopnea syndrome], peripheral vascular disease (demonstrated atherosclerotic disease in all arteries other than coronary arteries and aorta), cerebrovascular disease, any degree of cognitive impairment, any degree of functional disability, ischemic origin of reduced ejection fraction, previous HF admission, LVEF, and NYHA class I or II (vs. III, IV, or not available) at follow-up. All variables with a *p* value < 0.2 were entered into a multivariate binary logistic regression model, which served to estimate the PS of every patient. Patient matching was performed at a 1:1 ratio with the nearest neighbour method (caliper = 0.2 × standard deviation (SD) [logitPs]). Results are expressed as HR and 95% CI. Statistical analyses were performed with SPSS version 22.0 (SPSS Inc., Chicago, IL, USA).

## 3. Results

### 3.1. Study Population

A total of 364 patients were recruited. The mean age was 84.1 years (SD ± 5.2), of which 67% were male. A total of 81% were hypertensive and 33.5% diabetic. The main cause of ventricular dysfunction was ischemic, with 50.2%. At inclusion, 55.8% were in NYHA functional class II and the mean LVEF was 29.8% (SD ± 7.6). Almost half of the patients had chronic kidney disease, defined as estimated glomerular filtrate rate (eGFR) < 60 mL/min/1.73 m^2^ (47.8%). In terms of treatments, 81.6% were on beta-blockers, 51.6% on ACE inhibitors, 42.9% on aldosterone antagonists, 26.4% on ARNIs, and only 15.1% on SGLT2i (Table 1a).

### 3.2. Outcomes

Median follow-up was 33 months (SD ± 12.2) and there were a total of 122 (33.5%) deaths. 114 deaths occurred in the group of patients without SGLT2i (36.9%), while 8 deaths occurred in the group of patients treated with SGLT2i (14.5%). Regarding the cause of death, 23 were related with a cardiovascular event and 70 were not related with cardiovascular causes. The cause of death could not be clarified in 23.7% of the cases. Additionally, 85 hospitalizations for heart failure were registered in the overall population: 68 in patients without SGLT2i and 17 in patients with SGLT2i.

We performed a survival analysis in order to determine the significant predictors of total mortality, following the methodology described above. After a multivariate Cox logistic regression test for all-cause mortality, we observed that only SGLT2i (HR 0.39 [0.19–0.82] *p =* 0.014) and glomerular filtration rate (HR 0.98 [0.98–0.99] *p =* 0.026) proved to be protective factors. To this end, we performed another multivariate Cox regression analysis to determine the significant predictors of cardiovascular events (HF hospitalizations and cardiovascular mortality). Neither SGLT2i nor other HFrEF-specific treatments showed significant benefit for the CV outcomes in our population. Table 2 and Table 3 show the results of a univariate and multivariate analysis of all-cause mortality (Table 2) and CV events (Table 3).

In parallel, we conducted a propensity-score (PS)-matched analysis, in order to specifically determine the effect of SGLT2i in our population, according to the methodology previously described. After PS-matching, we selected 90 patients (45 in each group), whose baseline characteristics are shown in Table 1b. This PS analysis showed a significant reduction in all-cause mortality associated with the use of SGLT2i treatment (HR 0.39 (0.16–0.97) *p =* 0.04). However, similar to the multivariate Cox regression analysis, we found no significant relationship between iSGLT2 use and total CV events (death or HF admission), with a HR 0.78 (0.42–1.47).

**Figure 1:**
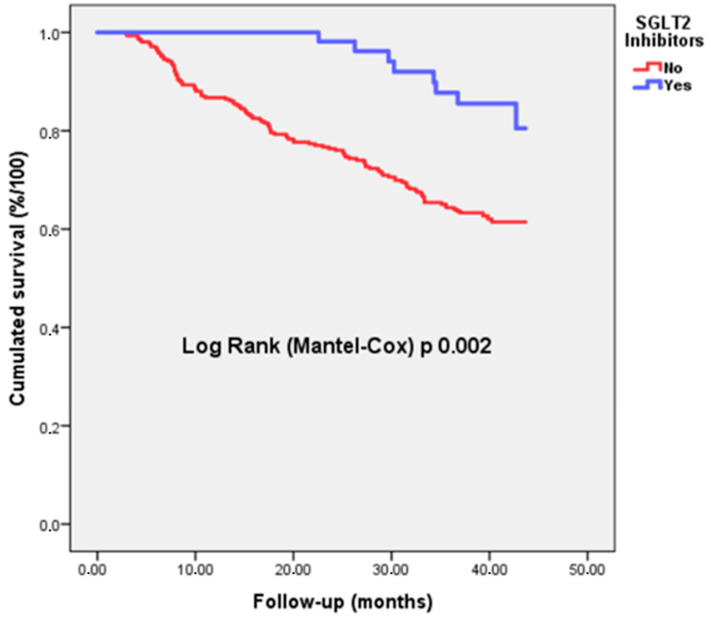
Kaplan–Meier survival curves showing unadjusted cumulative probability of all-cause death in patients aged >75 years with HFrEF, with and without SGLT2i treatment.

Kaplan–Meier curves for all-cause mortality, comparing the population under treatment with SGLT2i to those not treated with SGLT2i, are shown in Figure 1.

Through the study of electronic medical records, we investigated the reasons for the non-use of SGLT2i in our population. The main cause of non-treatment with SGLT2i was chronic kidney disease (4.9%), followed by hypotension (1.3%) and recurrent urinary tract infections (UTIs) (0.3%). This means that 91.6% of patients in our population without SGLT2i treatment had no clear contraindication for SGLT2i use.

## 4. Discussion

Heart failure presents a high prevalence in elderly patients. Those > 70 years have a tenfold higher HF prevalence than those under 55, and represent 50% of all patients with heart failure with reduced ejection fraction (HFrEF) [9]. The therapies used in daily practice have demonstrated clinical benefits on elderly patients, although several precautions must be observed due to the lower tolerability, impaired efficacy and reduced safety of these drugs in older patients. Lipid-soluble beta-blockers (e.g., metoprolol) present an increased penetrance in the elderly, associated with central nervous side effects (e.g., fatigue) [10]. ACEi, ARB, Mineralocorticoid Receptor Antagonists (MRA) and ARNI might require the monitoring of kidney functions and electrolytes, and slow dose titration to mitigate adverse effects. All these recommendations, among other factors, determine a lower rate of use of treatment in the elderly, with demonstrated clinical benefits. To this end, the CHAMP-HF registry showed the current limitations when titrating medication, with up to one third of patients without the dose recommended by the clinical practice guidelines of beta-blockers or ACEi/ARB/ARNI, and up to 67% of MRA, despite one year of following up [11,12]. Opposing this trend, there are data showing that the optimization of HF treatment in elderly patients is associated not only with a reduced risk of complications, but also with a reduction in related costs. In a population-based study in Spain of 19,762 HF patients with a mean age of 78.3 years, 76% of the cost was due to cardiovascular hospitalizations, in particular HF hospitalizations, and only 7% due to medication [13]. In spite of this, lower prescription rates in the elderly population are a reality in our routine clinical practice, and it may result from concerns about polypharmacy, lower tolerability, and the reduced safety or impaired efficacy of these drugs in older patients, among other factors [2].

On the other hand, in the last five years, the HF benefits of SGLT2i have been largely described. Initially, the CANVAS, EMPA-REG and DECLARE-TIMI 58 trials showed cardiovascular benefits (including HF hospitalizations) in diabetic patients [14,15,16,17,18,19,20,21,22,23,24,25,26]. Subsequently, specific trials were designed to assess the benefit of SGLT2i in patients with HFrEF. The EMPEROR-Reduced and DAPA-HF trials were two randomized, double-blind studies, in which 3730 and 4744 patients were included. Researchers evaluated strong clinical endpoints with significant results, such as CV mortality, mortality of any cause, urgent hospital visit with IV treatment for HF, and HF hospitalizations [4,5]. Furthermore, patients treated with empagliflozin presented clinical benefits compared with placebo on patients with acute hospitalization for HF in regardless of EF. The EMPULSE clinical trial obtained positive results for a hierarchical composite of death from any cause, number of HF events and time to first HF event, or an improvement in a quality of life score, where more patients with an acute hospitalization for HF, treated with empagliflozin, saw clinical benefits compared with placebo, regardless of LVEF [17]. Following this, the ESC guidelines included SGLT2i in 2021 as part of the recommended treatment of HFrEF. In addition, SGLT2i have recently shown significant clinical benefit in HFpEF as well, as shown at the EMPEROR-Preserved and DELIVER trials [18]. The former demonstrated a robust improvement in the combined endpoint of cardiovascular death and heart failure admission, mainly driven by a reduction in HF hospitalizations [19]. One year later, the DELIVER study showed a significant benefit in the composite of cardiovascular death and worsening heart failure (HF hospitalization or urgent HF visit), albeit no reduction in cardiovascular death [20]. Finally, a post hoc analysis suggests a potential benefit irrespective of age in patients with HF, with EF > 40%, although further evidence is required [21]. In light of the mentioned evidence, a recent focused update was published in August 2023 that increased the class of recommendations for SGLT2i in patients with HF with EF > 40%.

The available data on the benefit of SGLT2i in the very elderly with HFrEF are limited. One of the main causes is a common underrepresentation of elderly patients in trials. Dapagliflozin and empagliflozin trials represent a highly screened population, limiting the generalizability of their findings to real-world populations. To this end, Thorvaldsen et al. performed in a real-world outpatient HF setting, based on the Swedish HF Registry, where the eligibility criteria ranged between 30% and 35% when the inclusion and exclusion criteria of the main trials with dapagliflozin and empagliflozin were applied [22]. Regarding the use of SGLT2i in elderly with HF, Damman et al. demonstrated a significant reduction in the composite of admission for HF, all-cause mortality, and worsening HF during admission, as well as an association with a better response to diuretics, with no increase in side effects in a population in patients with a median age of 76 years [23]. Other exploratory post hoc analyses have been performed with contradictory results. Martinez et al. published a subanalysis from a DAPA-HF trial, with four subgroups of age, where a significant reduction in HF hospitalization or urgent HF visit and an improvement in KCCQ was observed in the very elderly (1149 patients > 75 years), with no improvement in mortality (HR 0.83 IC95% [0.58–1.17]) [24]. On the other hand, Fillipatos et al. performed a subanalysis from EMPEROR-Reduced trial with 999 patients older than 75 years old with HFrEF, where no CV benefits were found for this subgroup [25].

In our study we analyzed the outcomes of the use of SGLT2i in a population of very elderly patients (>75 years) with HFrEF. Our group conducted a real-world, retrospective, observational study, where SGLT2i significantly reduced all-cause mortality after a mean follow up of 33 months, although it did not reduce cardiovascular events, in relation to a high rate of admission for HF in both groups. We consider them highly clinically relevant for several reasons: first, our data show a significant benefit in this subgroup of patients with advanced age, commonly found in routine clinical practice, but far removed from the usual trial populations; second, our study presents a large number of very elderly patients with a prolonged follow-up, compared with the DAPA-HF and EMPEROR-Reduced subanalysis studies of just 16 and 18.2 months, respectively; third, due to the high frailty and comorbidity, we consider it unlikely that a future randomized trial with SGLT2i will be designed specifically in this subgroup of patients.

However, the high percentage of patients in the no-SGLT2i group, without a clear cause, is noteworthy. Until this publication, the available evidence on the efficacy and safety of this drug in this subgroup of patients was very scarce. One potential explanation is due to greater intolerance or comorbidities in this patient profile and a fear of adverse events. There have been reports of weight loss with the use of SGLT2i, largely driven by reducing body fat. This fat loss effect could be particularly relevant in elderly patients with increased frailty [26]. Clinically relevant weight reduction (considered as 5% of weight reduction) could increase by 23% with this treatment [27]. In a similar line, a risk of fracture increase was shown with canagliflozin [14], but not with empagliflozin or dapagliflozin [28]. Conversely, by means of a chronic caloric deficit state, SGLT2i might increase insulin sensitivity, favoring the anabolism of muscle tissue [29] and inducing body composition improvements through a reduction in fat mass with minor changes in lean mass [30]. UTIs could be another limiting factor for the use of SGLT2i in elderly patients, since urinary incontinence increases with age and UTIs are more frequent in diabetic patients [31,32]. In spite of this, SGLT2i therapy did not increase the incidence of UTI or genital infection events in trials with elderly individuals [25,29]. Moreover, SGLT2i have consistently demonstrated a nephroprotective capacity in both diabetic and non-diabetic patients [33]. Furthermore, DAPA-HF demonstrated in its study population an improved tolerability of full-dosage tolerance, with just 13.8% of the patients requiring dose reduction or an interruption of SGLT2i treatment [6]. In line with these results, in our study, few participants discontinued medication due to this adverse effect. SGLT2i are well tolerated by type 2 diabetic patients because of their low risk of hypoglycemia (<1%) in older adults, as shown in a metanalysis by Palmer et al., in which no difference of serious hypoglycemia was found in a comparison of SGLT2i treatment with a placebo [29,34]. Despite all of the above, the percentage of non-use of SGLT2i in our population of elderly patients with HFrEF was very high. A specific analysis of the causes of non-use was performed, showing that practically 90% of the population in which SGLT2i had not been initiated did not have a justified cause in their clinical history, which indicates the significant lack of adherence to ESC treatment recommendations in this subgroup of patients, including our setting [7]. This contrasts with the clinical benefit, in terms of mortality, that we observed in our study. Greater adherence to current treatment recommendations for HFrEF in elderly patients could improve prognosis in these patients.

Our study presents certain limitations. It is a retrospective, non-randomized study using a historical cohort from a single center. In addition, the study population is relatively small, which may affect the statistical results. A further limitation is that we could not determine the cause of death in 29 patients (23.7%) because it was not included in the available information.

## 5. Conclusions

In summary, the treatment of HFrEF remains a significant challenge in managing HF patients, especially in the elderly. According to our data, treatment with SGLT2i in elderly patients with HFrEF was associated with a lower rate of all-cause mortality. Our data show us that SGLT2i therapy could improve prognosis in the elderly with HFrEF in a real-world study. Nevertheless, the rate of undertreatment with SGLT2i in this subgroup of patients is very high. Adherence to the current treatment recommendations for the elderly could improve their prognosis, with a management focused on the progressive titration of main therapeutic lines, individualized treatment, and the control of the associated comorbidities in the affected individuals, where SGLT2i may play a main role due to the limited possibility of adverse events and its prognostic benefit.

## Figures and Tables

**Table 1:** (**a**) Characteristics of the patients at baseline. Quantitative data are presented as mean (standard deviation). Qualitative data are presented as percentages. (**b**) Characteristics of the patients at baseline after propensity-score matching. Quantitative data are presented as mean (standard deviation). Qualitative data are presented as percentages.

**(a)**			
**Variables**	**Total Population (N = 364)**		
Age, years (SD)	84.1 (5.2)		
Male sex, n (%)	244 (67)		
Hypertension, n (%)	295 (81)		
Diabetes, n (%)	122 (33.5)		
Hyperlipidaemia, n (%)	206 (56.6)		
Current smoker, n (%)	152 (41.8)		
BMI > 30 kg/m^2^, n (%)	48 (13.2)		
Lung disease, n (%)	59 (16.2)		
Stroke/TIA, n (%)	61 (16.8)		
Peripheral artery disease, n (%)	40 (11.0)		
Partially/Totally dependent on BADL (%)	38 (10.4)		
Ischemic LVSD, n (%)	167 (45.9)		
Previous HF admission, n (%)	142 (39)		
QRS width, ms (SD)	128 (34)		
Bundle brunch block	208 (57.1)		
LVEF, % (SD)	29.8 (7.6)		
Sinus rhythm, %	228 (62.6)		
Atrial fibrillation, %	125 (34.3)		
Paced rhythm	76 (20.8)		
CRT, n (%)	49 (13.5)		
NYHA class III or IV, %	42 (11.5)		
CKD (eGFR < 60 mL/min/1.73 m^2^), n (%)	173 (48.7)		
eGFR, mL/min/1.73 m^2^(SD)	59.6 (17.2)		
Stroke/TIA, %	61 (16.8)		
Betablockers, n (%)	297 (81.6)		
ACEi/ARB, n (%)	188 (51.6)		
ARNI, n (%)	156 (42.9)		
MRA, n (%)	96 (26.4)		
SGLT2i	55 (15.1)		
Diuretics, n (%)	262 (72)		
Nitrates, n (%)	27 (7.4)		
Digoxin, n (%)	27 (7.4)		
Antiplatelet therapy, n (%)	138 (37.9)		
OAC, n (%)	203 (55.8)		
Ivabradine, n (%)	14 (3.8)		
Amiodarone, n (%)	44 (13.5)		
**(b)**			
**Variables**	**SGLT2i (after Propensity-Score Matching) (N = 90)**		
	**No (N = 45)**	**Yes (N = 45)**	***p*** **Value ^a^**
Age, years (SD)	81.9 (5.1)	81.1 (4.6)	NS
Male sex, n (%)	35 (77.8)	35 (77.8)	NS
Hypertension, n (%)	40 (88.9)	38 (84.4)	NS
Diabetes, n (%)	20 (44.4)	23 (51.1)	NS
Hyperlipidaemia, n (%)	31 (68.9)	28 (62.2)	NS
Current smoker, n (%)	21 (46.7)	20 (44.4)	NS
BMI > 30 kg/m^2^, n (%)	6 (13.3)	4 (8.9)	NS
Lung disease, n (%)	5 (15.6)	5 (11.1)	NS
Stroke/TIA, n (%)	8 (17.8)	9 (20)	NS
Peripheral artery disease, n (%)	3 (6.7)	4 (8.9)	NS
Partially/Totally dependent on BADL (%)	5 (11.1)	0 (0.0)	0.027
Ischemic LVSD, n (%)	34 (75.6)	33 (73.3)	NS
Previous HF admission, n (%)	11 (24.4)	17 (37.8)	NS
QRS width, ms (SD)	122 (29.6)	134 (29.7)	NS
LVEF, % (SD)	27.1 (8.0)	28.4 (7.1)	NS
Sinus rhythm, %	24 (53.3)	37 (82.2)	0.006
Heart rate, bpm (SD)			
CRT, n (%)	7 (15.6)	9 (20)	NS
NYHA class III or IV, %	9 (20)	11 (24.4)	NS
CKD (eGFR < 60 mL/min/1.73 m^2^), n (%)	16 (35.6)	16 (35.6)	NS
eGFR, mL/min/1.73 m^2^ (SD)	64.2 (19.9)	62.5 (17.0)	NS
Betablockers, n (%)	35 (77.8)	42 (93.3)	0.032
ACEi/ARB, n (%)	21 (46.7)	12 (26.7)	0.048
ARNI, n (%)	10 (22.2)	32 (71.1)	0.000
MRA, n (%)	21 (46.7)	23 (51.1)	NS
SGLT2i	0 (0.0)	45 (100)	-
Diuretics, n (%)	27 (60)	34 (75.6)	NS
Nitrates, n (%)	4 (8.9)	3 (6.7)	NS
Digoxin, n (%)	2 (4.4)	1 (2.2)	NS
Antiplatelet therapy, n (%)	21 (46.7%)	28 (62.2%)	NS
OAC, n (%)	24 (53.3)	19 (42.2)	NS
Ivabradine, n (%)	2 (4.4)	1 (2.2)	NS
Amiodarone, n (%)	10 (22.2)	6 (13.3)	NS

ACE-I: angiotensin-converting enzyme inhibitors. ARNI: angiotensin receptor neprilysin inhibitor. ARB: angiotensin receptor blocker. BADL: basic activities of daily living. BMI: body-mass index. CKD: chronic kidney disease. COPD: chronic obstructive pulmonary disease. CRT: cardiac resynchronization therapy. eGFR: estimated glomerular filtration rate. HF: heart failure. LVEF: left ventricular ejection fraction. LVSD: left ventricular systolic dysfunction. MRA: mineralocorticoid receptor antagonists, NYHA: New York Heart Association. OAC: oral anticoagulant therapy. TIA: transitory ischemic attack. SGLT2i: Sodium-glucose cotransporter type 2 inhibitors. ^a^ Comparisons between groups (analysis of variance for quantitative and linear-association χ^2^ test for qualitative variables).

**Table 2:** Univariate and multivariate Cox logistic regression test for all-cause mortality.

	Univariate Analysis	Multivariate Analysis
Variables	HR (CI 95%) ^a^	HR (CI 95%) ^a^
**Age (years)**	**1.05 (1.02–1.09)**	**1.06 (1.02–1.09)**
**Male sex**	**1.72 (1.13–2.61)**	**1.78 (1.15–2.73)**
Stroke/TIA	1.48 (0.96–2.30)	1.58 (1.01–2.48)
**eGFR (mL/min/1.73 m^2^)**	**0.98 (0.97–0.99)**	**0.98 (0.97–0.99)**
**LVEF improvement**	0.70 (0.49–1.02)	NS
**Non-Sinus rhythm**	1.76 (1.23–2.52)	**1.68 (1.17–2.42)**
Bundle brunch block	1.14 (0.98–2.05)	NS
Betablockers	0.75 (0.49–1.16)	NS
ARNI	0.67 (0.43–1.04)	NS
**SGLT2i**	**0.33 (0.16–0.69)**	**0.39 (0.19–0.82)**

ARNI: angiotensin receptor neprilysin inhibitor. eGFR: estimated glomerular filtration rate. HR: Hazard ratio. LVEF: left ventricle ejection fraction. NS: not significant. TIA: transitory ischemic attack. SGLT2i: Sodium-glucose cotransporter type 2 inhibitors. ^a^ Bold value denote *p* value < 0.05.

**Table 3:** Univariate and multivariate Cox logistic regression test for cardiovascular events (HF hospitalizations and all-cause mortality).

	Univariate Analysis	Multivariate Analysis
Variables	HR (CI 95%) ^a^	HRR (CI 95%) ^a^
**Age (years)**	**1.03 (1.00–1.06)**	**1.03 (1.00–1.06)**
Male sex	1.37 (0.97–1.94)	NS
**Stroke/TIA**	**1.45 (0.99–2.13)**	**1.50 (1.01–2.22)**
NYHA	1.39 (0.90–2.15)	NS
**eGFR (mL/min/1.73 m^2^)**	**0.98 (0.97–0.99)**	**0.98 (0.97–0.99)**
**Non-Sinus rhythm**	**1.69 (1.25–2.30)**	**1.70 (1.24–2.33)**
CRT	1.08 (0.70–1.67)	NS
Betablockers	0.89 (0.60–1.31)	NS
ACEI/ARB	0.77 (0.57–1.05)	NS
SGLT2i	0.80 (0.51–1.25)	NS

ACEI: angiotensin-converting enzyme inhibitor. ARB: angiotensin receptor blockers. ARNI: angiotensin receptor neprilysin inhibitor, BADL: basic activities of daily living. CKD: chronic kidney disease. CRT: cardiac resynchronization therapy. eGFR: estimated glomerular filtration rate. LVEF: left ventricular ejection fraction. NS: not significant. NYHA: New York Heart Association. TIA: transitory ischemic attack. SGLT2i: Sodium-glucose cotransporter type 2 inhibitors. ^a^ Bold value denote *p* value < 0.05.

## Data Availability

The data presented in this study are available on request from the corresponding author.
